# Association of hospital volume and operative approach with clinical and financial outcomes of elective esophagectomy in the United States

**DOI:** 10.1371/journal.pone.0303586

**Published:** 2024-06-14

**Authors:** Saad Mallick, Nikhil L. Chervu, Jeffrey Balian, Nicole Charland, Alberto R. Valenzuela, Sara Sakowitz, Peyman Benharash

**Affiliations:** 1 Cardiovascular Outcomes Research Laboratories (CORELAB), David Geffen School of Medicine, University of California, Los Angeles, CA, United States of America; 2 Department of Surgery, David Geffen School of Medicine, University of California, UCLA, Los Angeles, CA, United States of America; Public Library of Science, UNITED STATES

## Abstract

**Introduction:**

Literature regarding the impact of esophagectomy approach on hospitalizations costs and short-term outcomes is limited. Moreover, few have examined how institutional MIS experience affects costs. We thus examined utilization trends, costs, and short-term outcomes of open and minimally invasive (MIS) esophagectomy as well as assessing the relationship between institutional MIS volume and hospitalization costs.

**Methods:**

All adults undergoing elective esophagectomy were identified from the 2016–2020 Nationwide Readmissions Database. Multiple regression models were used to assess approach with costs, in-hospital mortality, and major complications. Additionally, annual hospital MIS esophagectomy volume was modeled as a restricted cubic spline against costs. Institutions performing > 16 cases/year corresponding with the inflection point were categorized as high-volume hospitals (HVH). We subsequently examined the association of HVH status with costs, in-hospital mortality, and major complications in patients undergoing minimally invasive esophagectomy.

**Results:**

Of an estimated 29,116 patients meeting inclusion, 10,876 (37.4%) underwent MIS esophagectomy. MIS approaches were associated with $10,600 in increased incremental costs (95% CI 8,800–12,500), but lower odds of in-hospital mortality (AOR 0.76; 95% CI 0.61–0.96) or major complications (AOR 0.68; 95% CI 0.60, 0.77). Moreover, HVH status was associated with decreased adjusted costs, as well as lower odds of postoperative complications for patients undergoing MIS operations.

**Conclusion:**

In this nationwide study, MIS esophagectomy was associated with increased hospitalization costs, but improved short-term outcomes. In MIS operations, cost differences were mitigated by volume, as HVH status was linked with decreased costs in the setting of decreased odds of complications. Centralization of care to HVH centers should be considered as MIS approaches are increasingly utilized.

## Introduction

Despite advances in surgical techniques and perioperative management, esophagectomy remains a complex operation that can result in significant morbidity [1,2]. Although recent literature has suggested MIS esophagectomy abbreviates inpatient stay, associations with postoperative complications have been controversial [3–7]. In a study of more than 10,000 patients undergoing esophagectomy, Khaitan et al reported higher rates of anastomotic leak, pulmonary embolism, and reoperation associated with MIS approaches [4].

Quality of care aside, healthcare systems and payers have been evaluating strategies to curb costs of care. Although MIS now represents the predominant approach to esophagectomy among Society of Thoracic Surgeons (STS) reporting centers, the cost-efficiency of such methods continue to be debated [4]. Proponents of MIS argue that associated reductions in length of stay, wound care and disability, theoretically translate to lower net expenditures [3,8,9]. Recent adoption of robotic platforms further nuances this debate, as robot acquisition and auxiliary disposable equipment incur significant costs. Although prior analyses have examined patient and hospital-level drivers of esophagectomy cost, the impact of surgical approach has not been reported [10,11]. Previous studies describing cost-effectiveness of esophagectomy at high-volume centers have not factored surgical approach into their analyses [11,12].

The present work used a nationally representative cohort to examine temporal trends in use, in-hospital costs, and short-term outcomes of open and MIS (laparoscopic, thoracoscopic, robotic) esophagectomy. We hypothesized MIS to be associated with increased index costs of care but comparable in-hospital mortality, major complications, and 30-day readmissions, compared to the open approach. We further theorized that increasing operative volume would mitigate the excess cost associated MIS esophagectomy.

## Methods

### Data source and cohort definitions

**Fig 1:**
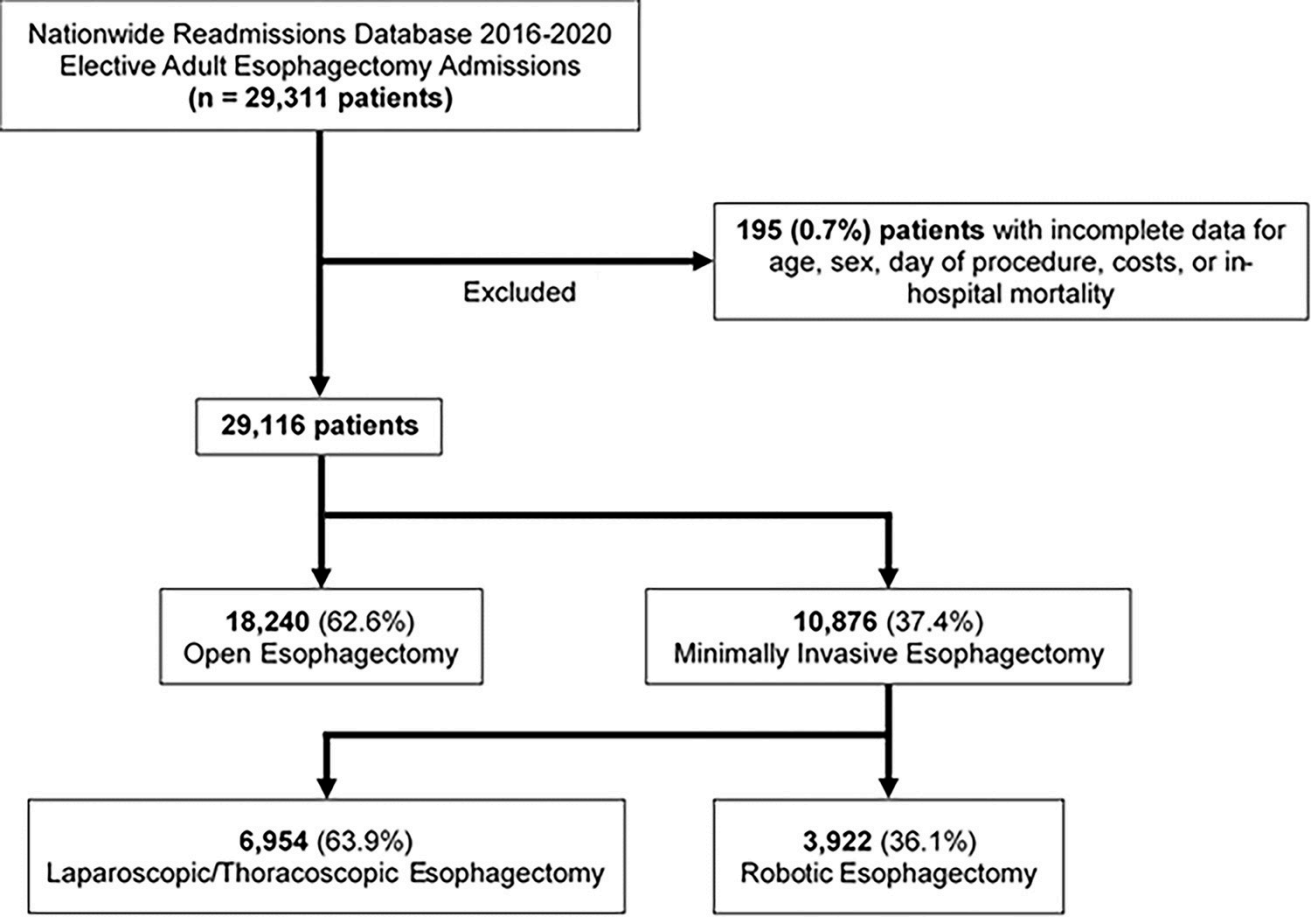
Exclusion criteria.

This was a retrospective cohort study of the 2016 to 2020 Nationwide Readmissions Database (NRD). The NRD uses survey weighting methodology to provide accurate estimates for ~60% of all hospitalizations in the United States [13]. Unique patient linkage numbers allow for tracking of readmissions across hospitals during each calendar year. Using relevant *International Classification of Diseases Code*, *Tenth Revision* (ICD-10) codes, all elective adult (≥18 years of age) hospitalizations for esophagectomy were identified (S1 Table). Additionally, ICD-10 diagnosis codes were used to identify malignant conditions (S2 Table), which were accounted for in risk adjusted-models. Records missing data for age, sex, day of procedure, charges, or in-hospital mortality were excluded from further analysis (0.7%, Fig 1).

### Variable definitions and study outcomes

Patient and hospital characteristics including age, sex, income quartile, primary payer, urbanicity, and teaching status, were defined according to the NRD data dictionary [13]. The van Walraven modification of the Elixhauser Comorbidity Index was used to quantify burden of chronic conditions [14,15]. Index hospitalization costs were calculated by applying cost-to-charge ratios (CCRs) to overall charges, with inflation adjustment to the 2020 Personal Health Index [16,17]. The NRD provided CCRs are specific to individual hospitals, hospital systems, and peer groups, and allow for the estimation of the total costs of each patient stay. The NRD has developed a methodology for estimating hospital inpatient costs based on the cost reports provided by hospitals which include direct costs such as costs of inpatient, outpatient, and ancillary services, and indirect costs such as general, administrative, and capital contributions [18]. Additionally, the NRD is sampled such that nationally representative estimates of hospital costs and readmission costs can be derived using the hospital-specific weights, strata and cluster variables [16].

The primary outcome of interest was index hospitalization costs. Secondary endpoints included in-hospital mortality, major complications, postoperative duration of stay (pLOS), and 30-day non-elective readmissions. Major complications were defined in accordance with Society of Thoracic Surgeons (STS) guidelines and encompassed perioperative stroke or transient ischemic attack (TIA), prolonged ventilation (>96 hours), acute renal failure requiring dialysis, and reoperation [19].

Patients undergoing open esophagectomy comprised the *Open* cohort, while laparoscopic, thoracoscopic and robotic esophagectomy patients were classified as *MIS*. Annual hospital MIS esophagectomy volume was defined as the total number of MIS esophagectomy cases performed at each center. This volume was modeled as a restricted cubic spline in regression models to allow for nonlinear relationships. Hospitals with an annual volume at or above the cost-volume inflection point were categorized as high-volume (HVH), while all others were classified as low-volume (LVH).

### Statistical analysis

The significance of temporal trends was determined using a non-parametric rank-based test developed by Cuzick [20]. Continuous variables are reported as medians with interquartile range (IQR), while categorical variables are shown as proportions (%). The Adjusted Wald and Pearson’s χ^2^ tests were used to assess the significance of intergroup differences for continuous and categorical variables, respectively. We employed inverse probability of treatment weighing (IPTW) to account for differences in patient and hospital characteristics between those who underwent MIS esophagectomy and their counterparts. This approach utilizes weights derived from propensity scores, enabling the control of potential confounders without diminishing the original sample size [21]. To confirm that reweighting resulted in balanced populations, the standardized mean differences in baseline characteristics of patients before and after IPTW were compared [22]. Following application of IPTW, logistic regression models for binary outcomes and linear models for continuous outcomes, were developed to evaluate the independent association between operative approach and outcomes of interest. Regression outputs are reported as adjusted odds ratios (AOR) for discrete and beta coefficients (β) for continuous variables, both with 95% confidence intervals.

Statistical analysis was performed using Stata 16.0 (StataCorp LLC, College Station, TX) with significance set at α = 0.05. This study was deemed exempt from full review by the Institutional Review Board at the University of California, Los Angeles. Authors did not have access to information that could identify individual participants during the study due to deidentified nature of the NRD.

## Results

### Time trends in the use of MIS Esophagectomy

**Fig 2:**
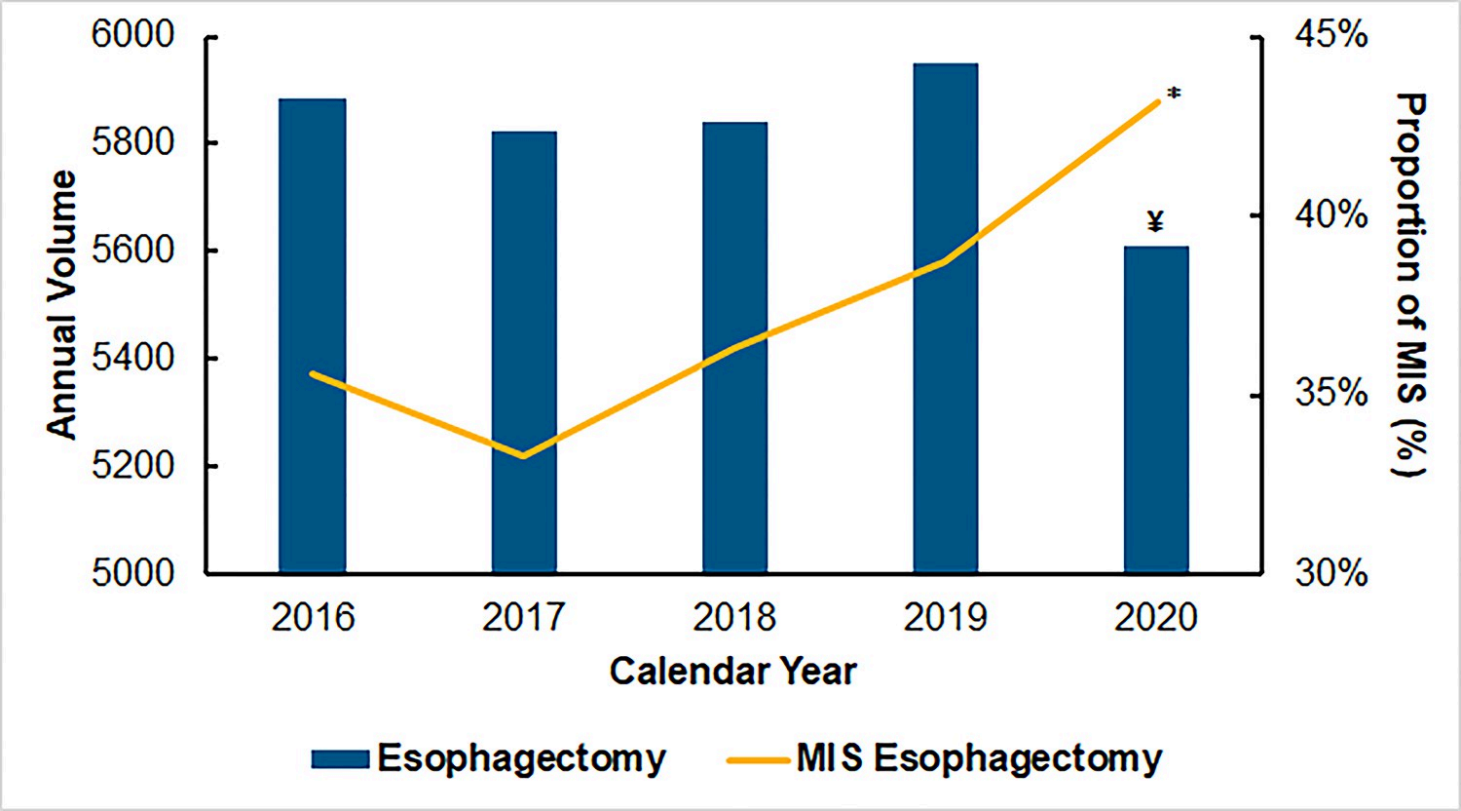
National trends in esophagectomy volume and approach; MIS, minimally invasive surgery; *nptrend<0.001; ^¥^2020 = Covid-19 year.

**Fig 3:**
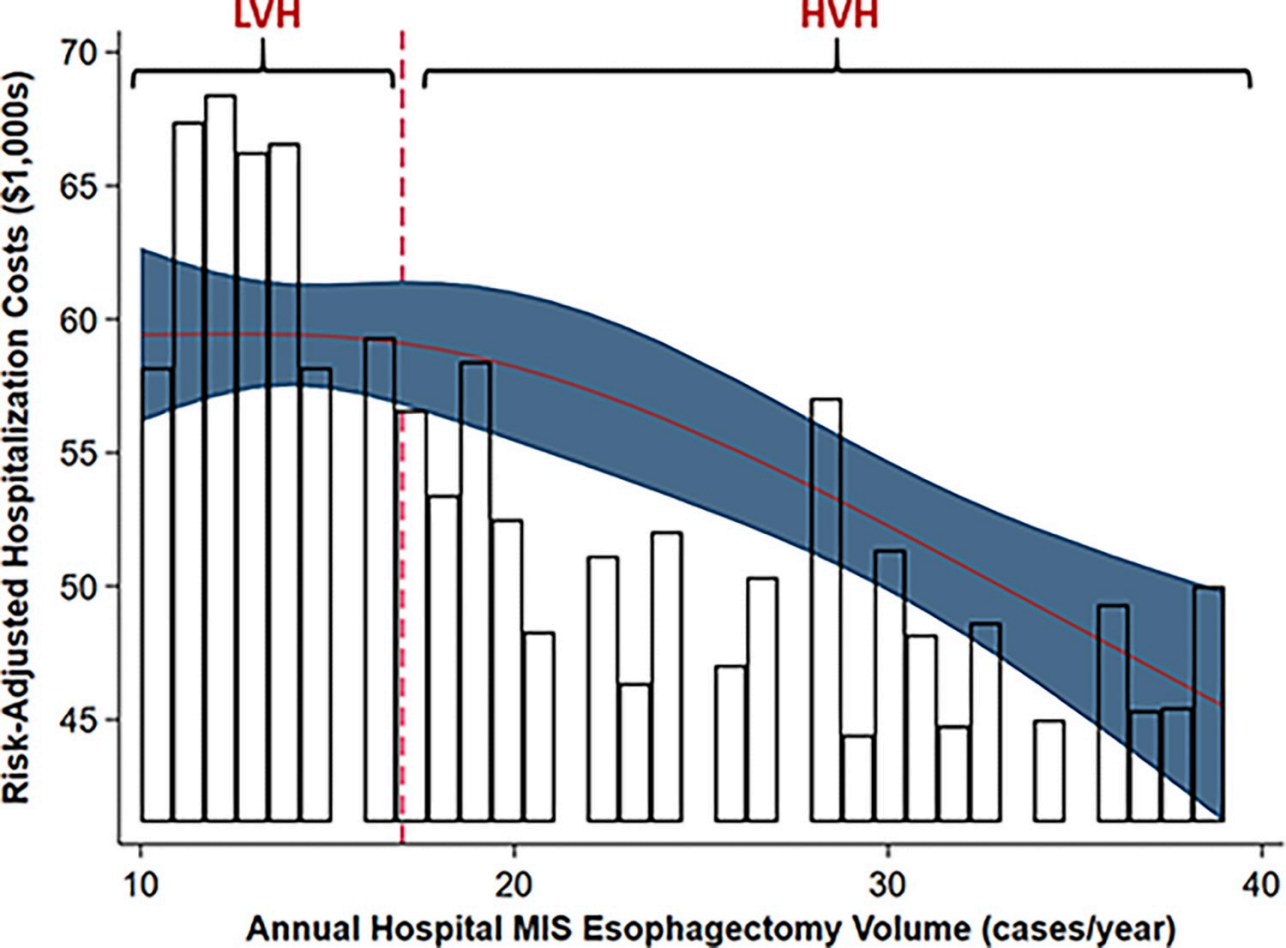
Association of annual hospital MIS esophagectomy volume and inpatient costs; MIS, minimally invasive surgery; LVH, low volume hospitals; HVH, high volume hospitals.

Of an estimated 29,116 patients undergoing esophagectomy during the study period, 10,876 (37.4%) underwent MIS esophagectomy. From 2016 to 2020, the proportion of MIS significantly increased from 35.6% to 43.2% (nptrend<0.001, Fig 2). Moreover, the proportion of MIS esophagectomy cases with robotic assistance increased from 29.2% to 40.1% (nptrend<0.001) during this epoch. Malignancy as the operative indication was present in 65.9% of *Open* and 73.8% of *MIS* patients. Additionally, the total number of NRD-participating hospitals performing MIS esophagectomy increased from 243 in 2016 to 266 in 2020, with a median annual caseload of 11 (IQR 6–16, nptrend<0.001). On risk-adjusted modeling of costs vs center-level MIS volume, an inflection point was noted at 16 cases/year (Fig 3). Based on this threshold, when looking at patients undergoing MIS alone, 14.4% of operating centers were considered HVH and 85.6% LVH.

### Patient characteristics and outcomes by Operative Approach

Detailed cohort characteristics are show in Table 1. Compared to *Open*, *MIS* patients were younger and had a lower Elixhauser Index. Subjects in the MIS group were more likely to belong to the highest income quartile (24.7 vs 19.7%, p<0.001) but were comparable in primary payer status. The *MIS* cohort was less likely to be managed at large hospitals (75.1 vs 80.4%, p = 0.01) but more likely to receive surgery in a metropolitan setting (93.5 vs 91.9%, p = 0.01). The cohorts were also similar in terms of sex distribution. On unadjusted analysis, *MIS* had similar median in-hospital costs to *Open* patients ($42,700 [IQR 28,500–63,200] vs. 38,900 [IQR 25,600–61,000]) despite shorter median pLOS (Table 2). However, *MIS* had lower rates of in-hospital mortality (2.1 vs 3.2%, p<0.001) and major complications (9.3 vs 14.5%, p<0.001) compared to *Open*. *MIS* patients experienced lower rates of all considered postoperative complications. Rate of unplanned rehospitalizations at 30 days following index discharge was similar among the two groups (Table 2).

**Table 1:** Demographic and hospital characteristics of patients undergoing open versus minimally invasive (MIS) esophagectomy from 2016–2020; IQR, interquartile range.

	Open (n = 18,240)	MIS (n = 10,876)	p-value
Age (years, median, IQR)	65 [57–71]	65 [57–72]	0.003
Female (%)	26.7	28.3	0.07
*Primary payer (%)*			0.06
Private	36.9	39.4	
Medicare	50.8	49.1	
Medicaid	7.8	7.2	
	1.2	1.1	
Other payer	0.2	3.1	
*Income quartile (%*, *Percentile)*			<0.001
0^th^-25th (lowest)	23.5	18.5	
26th-50th	29.2	28.8	
51st-75th	26.2	26.9	
76th-100th (highest)	19.7	24.7	
Elixhauser Index (median, IQR)	4 [2–5]	4 [2–5]	0.04
*Hospital setting (%)*			0.01
Large metropolitan areas	91.9	93.5	
Small metropolitan areas	6.5	5.9	
Not metropolitan	1.6	0.6	
*Hospital teaching status (%)*			0.09
Teaching	91.9	93.5	
Non-teaching	8.1	6.5	
*Bed size (%)*			0.01
Small	6.6	9.5	
Medium	13.0	15.0	
Large	80.4	75.0	
Malignant disease (%)	65.9	73.8	<0.001
*Comorbidities (%)*			
Cancer, metastatic	18.4	17.0	0.13
Cardiac arrhythmia	35.5	33.2	0.01
Chronic liver disease	5.0	5.8	0.10
Chronic lung disease	21.7	20.8	0.21
Coagulopathy	8.0	7.5	0.41
Congestive heart failure	4.9	4.1	0.03
Diabetes	20.4	21.0	0.48
End stage renal disease	6.5	5.9	0.26
Hypertension	57.3	58.0	0.45
Neurologic disorders	5.8	4.7	0.01
Obesity	15.5	20.2	<0.001
Peripheral vascular disease	5.3	5.7	0.33
Pulmonary hypertension	2.9	2.4	0.05

**Table 2:** Unadjusted perioperative outcomes for open versus minimally invasive (MIS) esophagectomy; TIA, transient ischemic attack; pLOS, postoperative length of stay; IQR, interquartile range.

	Open	MIS	p-value
	(n = 18,240)	(n = 10,876)	
In-Hospital Mortality (%)	3.2	2.1	<0.001
**Major Complications (%)**			
Stroke/TIA	0.6	0.2	0.001
Prolonged ventilation	6.2	3.5	<0.001
Acute renal failure requiring dialysis	9.7	7.2	<0.001
Reoperation	0.3	0.1	0.01
**Resource Utilization**			
pLOS (days, median, IQR)	9 [7 – 14]	6 [6 – 11]	<0.001
Costs ($1,000s, median, IQR)	38.9 [25.6–61.0]	42.7 [28.5–63.2]	0.61
30-day readmission (%)	13.5	12.9	0.41

**Fig 4:**
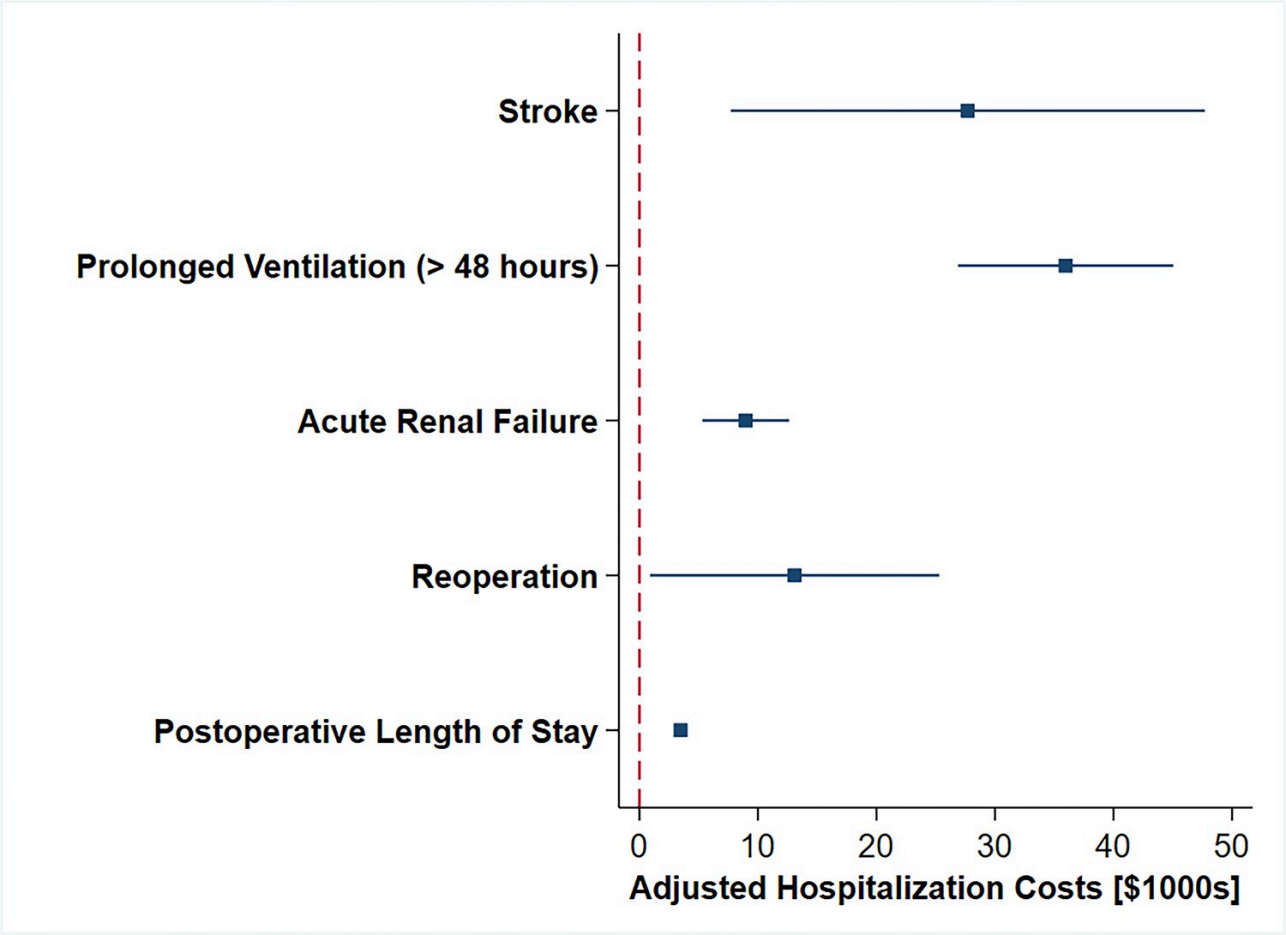
Perioperative factors associated with differences in adjusted hospitalization costs; all displayed factors are significantly associated with increased or decreased adjusted costs.

After IPTW adjustment, MIS was associated with a $10,600 increment in hospitalization costs (95% CI 8,800, 12,500, Ref: Open). Development of stroke/TIA, prolonged ventilation, acute renal failure, and reoperation were similarly associated with increased adjusted costs. Similarly, prolonged length of stay was also associated with increased hospitalization costs (Table 3 and Fig 4).

**Table 3:** Adjusted results of minimally invasive (MIS) approach on clinical outcomes and resource use following esophagectomy; AOR, adjusted odds ratio; CI, confidence interval; β, beta coefficient; TIA, transient ischemic attack; pLOS, postoperative length of stay.

	AOR or β Coefficient	95% CI	p-value
In-Hospital Mortality (AOR)	0.76	0.61, 0.96	0.02
**Major Complications (AOR)**			
Stroke/TIA	0.44	0.24, 0.80	0.01
Prolonged ventilation	0.63	0.52, 0.76	<0.001
Acute renal failure requiring dialysis	0.81	0.70, 0.93	0.003
Reoperation	0.30	0.10, 0.86	0.03
**Resource Utilization**			
pLOS (β, days)	-1.70	-2.16, -1.24	<0.001
Costs (β, $1,000s)	10.6	8.76, 12.47	0.03
30-day readmission (AOR)	0.97	0.87, 1.08	0.56

Deployment of MIS techniques was associated with significantly lower odds of in-hospital mortality (AOR 0.76; 95% CI 0.61, 0.96) and major complications (AOR 0.68; 95% CI 0.60, 0.77), with open approach as reference. MIS was specifically linked with reduced odds of prolonged ventilation, renal failure, stroke/TIA, and reoperation (Table 3). Despite increased adjusted costs, MIS utilization was associated with reduced pLOS (β -1.70 days; 95% CI -2.16, -1.24, Ref: open). Finally, MIS esophagectomy did not alter the likelihood of 30-day readmissions.

### Subgroup Analysis—Laparoscopic/Thoracoscopic vs Robotic Esophagectomy

Additional subgroup analysis was conducted to determine potential differences between laparoscopic/thoracoscopic and robotic esophagectomy. Of an estimated 10,876 MIS patients, 36.1% underwent robotic esophagectomy. Patients undergoing robotic surgery were comparable in age, Elixhauser Comorbidity Index scores, and sex distribution to their laparoscopic/thoracoscopic counterparts. Notably, recipients of robotic surgery were more likely to be in the lowest income quartile despite similar payer status. The proportion of patients undergoing esophageal resection for malignant disease were comparable between groups (S3 Table). Unadjusted clinical and financial outcomes were also similar and detailed in S4 Table.

After IPTW, robotic esophagectomy was associated with a $4,000 increment in index hospitalization costs (95% CI 800, 7100). Additionally, in-hospital mortality, major complications, pLOS, or 30-day readmission rates were comparable between surgical approaches after risk-adjusted analysis (S5 Table).

### Association of center volume with outcomes in MIS esophagectomy patients

When examining patients undergoing MIS exclusively, procedures conducted at HVH were associated with reduction of $6,600 in hospital expenditures (95%CI -12,000, -800, Ref: LVH). Using the established volume thresholds and LVH as reference, undergoing MIS at an HVH was associated with similar odds of in-hospital mortality (AOR 0.86; 95% CI 0.49, 1.53) and major complications (AOR 0.83; 95% CI 0.70, 0.96, p = 0.05). HVH were associated with lower odds of prolonged ventilation, and reoperation. No difference was noted in pLOS and 30-day readmission rates between HVH and LVH (Table 4).

**Table 4:** Adjusted results of high-volume status on clinical outcomes and resource use following MIS esophagectomy; AOR, adjusted odds ratio; CI, confidence interval; β, beta coefficient; TIA, transient ischemic attack; pLOS, postoperative length of stay.

	AOR or β Coefficient	95% CI	p-value
In-Hospital Mortality (AOR)	0.86	0.49, 1.53	0.61
**Major Complications (AOR)**			
Stroke/TIA	0.35	0.04, 2.81	0.32
Prolonged ventilation	0.50	0.35, 0.71	<0.001
Acute renal failure requiring dialysis	0.81	0.56, 1.23	0.32
Reoperation	0.30	0.10, 0.89	0.03
**Resource Utilization**			
pLOS (β, days)	-0.46	-1.68, 0.75	0.45
Costs (β, $1,000s)	-6.56	-12.03, -0.79	0.03
30-day readmission (AOR)	1.33	0.98, 1.61	0.08

## Discussion

To address the relative dearth of literature examining the economic implication of MIS esophagectomy, the present work utilized an all-payer, nationwide database to assess trends in utilization, costs, and short-term outcomes of such operations. While the adoption of minimally invasive surgical techniques continued to rise during our study period, open esophagectomy remained the dominant surgical approach. Despite increased costs, the MIS approach was associated with significantly lower odds of in-hospital mortality and complications. Finally, using LVH as reference, patients undergoing MIS at HVHs were associated with significantly lower costs, and fewer complications. Several of our findings warrant further discussion.

In the present work, we noted a significant rise in the use of MIS esophagectomy during the five-year study period. This finding mirrors trends observed across a wide range of surgical specialties including colon, lung, and pancreas resections [23,24]. In contrast to prior STS reports, open esophagectomy remained the dominant approach within our study population, comprising 56.8% of cases in 2020 [4]. In contrast, a 2020 analysis by Sheetz et al. found MIS to be the utilized in a majority of esophagectomy cases in the state of Michigan [25]. Such differences may be explained by study capture, as our results represent a nationwide sample inclusive of non-STS reporting institutions. The more gradual adoption of MIS in specific regions may be driven by relatively small numbers of annual resections, market competition, and surgical expertise [26]. Although MIS may ultimately benefit patients, the inherent complexity of minimally invasive techniques could decelerate utilization and present challenges to trainees [27,28]. Indeed, a 2022 systematic review noted significant variation in clinical outcomes amongst centers newly adopting robotic esophagectomy [29]. In addition to standardized training programs aimed at reducing such disparities, new advances in integrated data collection and real-time feedback may provide further benefit [30]. Given the increasing utilization of MIS and robotic approaches, development and analysis of standardized practice guidelines and dissemination of best practices are imperative to minimize variability in patient outcomes and operative efficiency.

Although MIS approaches were associated with improved short-term outcomes compared to open esophagectomy, they were linked to increased hospitalization costs. Specifically, laparoscopic/thoracoscopic and robotic approaches were associated with a respective $10,600 and $4,000 increase in adjusted costs, compared to open. The additional costs of robotic assistance may be explained by surgeon and staff training periods as well as increased operating room setup times [29]. A 2013 study by Lee et al. found MIS esophagectomy to be associated with greater operative expense but an annual cost decrement of $1,641, challenging our findings. This overall cost-saving was attributed to decreased length of stay and postoperative complications, a pattern consistent with findings by Parameswaran et al. Because the analysis of Lee and coworkers was limited to high-volume centers, operative cost may have been different compared to our inclusive population. Furthermore, the authors gleaned institutional costs from a primary center and extrapolated it to other centers, further limiting accurate cost assessment. Despite the observed increase in costs associated with MIS in our study, benefits to in-hospital mortality and complication rates may justify its use. In particular, the overall cost benefit of robotic approaches might not fully be captured by our study. Robotic pancreatectomy, for example, has shown to be superior regarding negative margin rate, organ preservation rate, and lower rates of blood loss [31–34]. Prospective examination of long-term measures including patient-reported outcomes may better clarify the value of MIS esophagectomy.

Notably, MIS operations performed at HVH were associated with significantly decreased costs compared to LVH, a likely consequence of reduced complications, streamlined hospital practices, and greater technical expertise. Goense et al previously reported a greater than €30,000 increase in total esophagectomy costs when postoperative complications were present [35]. Ho et al noted that high-volume surgeons were 10.6% less costly in comparison to their low-volume counterparts for esophagectomy [36]. In contrast, prior studies evaluating institutional volume and costs have found HVH to portend better outcomes at relatively similar costs to LVH [11,12]. While the Leapfrog Group, currently recommends 20 annual cases as the appropriate center-based threshold for esophagectomy, this varies from our defined threshold [37]. These differences may be attributed to our designation of HVH by MIS caseload rather than total esophagectomy cases and may indicate approach-specific volume thresholds as a more nuanced method to assess volume-outcome relationships. Nevertheless, our findings further support the notion that regionalization of MIS esophagectomy may benefit patient outcomes if minimum caseloads are not met. It should be noted that regionalization efforts have had mixed impact, with conflicting reports on the proportion of operations performed at HVH [12,38–41]. Notably, Finlayson et al showed that patients were less willing to travel for improved care and would rather stay near their home [42]. This choice may be primarily financial or logistical, with a more recent study showing that patients were willing to obtain higher quality care if barriers to travel could be addressed [43]. Directed strategies to facilitate HVH patient referrals by insurance companies may be necessary to improve patient outcomes and reduce systemic healthcare costs.

Our study has several limitations due to its use of an administrative database. The NRD lacks granularity in laboratory values, intraoperative events, and discussions that may affect patient-physician decision regarding surgical approach. Moreover, factors that may contribute to hospitalization costs such as operative time, blood loss, interval to oral feeding, and stage of cancer are not available in the NRD, and thus these could not be controlled for in our analysis. In addition, use of the NRD relies on accurate ICD coding which is primarily used for financial reimbursement. While cost to charge ratios provide a valuable insight into total hospitalization cost, we are unable to assess costs associated with preoperative and postoperative outpatient care. Moreover, operating room costs as well as long-term expenditure associated with the robotic system itself, including the upfront cost and amortization, are not captured separately delineated within the charge data provided by the NRD. Finally, we are unable to determine any causal relationships due to the retrospective nature of our study.

## Conclusions

Although increased adoption of MIS techniques for esophagectomy were noted, open esophagectomy remained the dominant approach nationwide. This is despite MIS being associated with improved adjusted in-hospital mortality and complication rates. Additionally, MIS is associated with significant increases in hospitalization costs, with robotic techniques demonstrating particularly high expenditures. Finally, high-volume esophagectomy hospitals were found to have improved outcomes at lower costs. These findings suggest that the centralization of care to HVH centers–especially in MIS esophagectomy– should be considered as a promising cost-reduction strategy.

## Supporting information

S1 Table International classification of diseases code, tenth revision (ICD-10) procedure codes for esophagectomy. (DOCX)

S2 Table Classification of diseases code, tenth revision (ICD-10) procedure codes for esophagectomy. (DOCX)

S3 Table Demographic and hospital characteristics of patients undergoing laparoscopic or thoracoscopic versus robotic esophagectomy from 2016–2020; IQR, interquartile range. (DOCX)

S4 Table Unadjusted perioperative outcomes for laparoscopic or thoracoscopic versus robotic esophagectomy; TIA, transient ischemic attack; pLOS, postoperative length of stay; IQR, interquartile range. (DOCX)

S5 Table Adjusted results of robotic approach on clinical outcomes and resource use following esophagectomy as compared to laparoscopic or thoracoscopic approaches; AOR, adjusted odds ratio; CI, confidence interval; β, beta coefficient; TIA, transient ischemic attack; pLOS, postoperative length of stay. (DOCX)

## Abbreviations

MIS: Minimally Invasive Surgery
HVH: High-volume hospitals
LVH: Low-Volume Hospitals
AOR: Adjusted Odds Ratio
CI: Confidence Interval
STS: Society of Thoracic Surgeons
NRD: Nationwide Readmissions Database
ICD-10: International Classification of Diseases Code, Tenth Revision
CCR: Cost-to-charge ratio
pLOS: postoperative length of stay
TIA: transient ischemic attack
IQR: interquartile range
IPTW: inverse probability of treatment weighing
β: beta coefficients
