# Supplementary material for: Association of hospital volume and operative approach with clinical and financial outcomes of elective esophagectomy in the United States
Source: PLoS One. 2024 Jun 14;19(6):e0303586. doi: 10.1371/journal.pone.0303586 (PMC11178205; doi:10.1371/journal.pone.0303586)
Supplement: S1 Table — (DOCX) [file pone.0303586.s001.docx]

**Supplementary Table 1:** International Classification of Diseases Code, Tenth Revision (ICD-10) procedure codes for esophagectomy

| **Category** | **ICD-10 Procedure Codes** |
| --- | --- |
| Open Esophagectomy | 0DB50ZZ, 0DB10ZZ, 0DB20ZZ, 0DB30ZZ, 0DT50ZZ, 0DT10ZZ, 0DT20ZZ, 0DT30ZZ, 0DR507Z |
| Minimally Invasive Esophagectomy (MIS) | 0DB54ZZ, 0DB14ZZ, 0DB24ZZ, 0DB34ZZ, 0DT54ZZ, 0DT14ZZ, 0DT24ZZ, 0DT34ZZ, 0DR547Z |
| Robotic Esophagectomy | Occurrence of a preceding code with one of the following codes: 8E0WXCZ, 8E0W0CZ, 8E0W4CZ |
