# Supplementary material for: Association of hospital volume and operative approach with clinical and financial outcomes of elective esophagectomy in the United States
Source: PLoS One. 2024 Jun 14;19(6):e0303586. doi: 10.1371/journal.pone.0303586 (PMC11178205; doi:10.1371/journal.pone.0303586)
Supplement: S2 Table — (DOCX) [file pone.0303586.s002.docx]

**Supplementary Table 2:** International Classification of Diseases Code, Tenth Revision (ICD-10) procedure codes for esophagectomy

| **ICD-10 Diagnosis Codes** | **ICD-10 Code Description** |
| --- | --- |
| C15 | Malignant neoplasm of esophagus |
| C16 | Malignant neoplasm of cardia |
| D00.1 | Carcinoma in situ of esophagus |
