# Supplementary material for: Association of hospital volume and operative approach with clinical and financial outcomes of elective esophagectomy in the United States
Source: PLoS One. 2024 Jun 14;19(6):e0303586. doi: 10.1371/journal.pone.0303586 (PMC11178205; doi:10.1371/journal.pone.0303586)
Supplement: S3 Table — (DOCX) [file pone.0303586.s003.docx]

**Supplementary Table 3:** Demographic and hospital characteristics of patients undergoing laparoscopic or thoracoscopic versus robotic esophagectomy from 2016-2020; IQR, interquartile range

|  | **Laparoscopic/Thoracoscopic**  **(n = 6,954)** | **Robotic**  **(n = 3,922)** | **p-value** |
| --- | --- | --- | --- |
| Age (years, median, IQR) | 65 [57 - 71] | 65 [57 - 71] | 0.62 |
|  |  |  |  |
| Female (%) | 29.0 | 27.1 | 0.17 |
|  |  |  |  |
| *Primary payer (%)* |  |  | 0.77 |
| Private | 40.0 | 38.4 |  |
| Medicare | 48.6 | 50.1 |  |
| Medicaid | 7.4 | 7.0 |  |
| Uninsured | 0.9 | 1.3 |  |
| Other payer | <0.1 | 0.1 |  |
|  |  |  |  |
| *Income quartile (%, Percentile)* |  |  | 0.04 |
| 0^th^-25th (lowest) | 17.3 | 20.6 |  |
| 26th-50th | 29.2 | 20.6 |  |
| 51st-75th | 26.6 | 27.5 |  |
| 76^th^-100th (highest) | 25.9 | 22.7 |  |
|  |  |  |  |
| Elixhauser Index (median, IQR) | 4 [2 - 5] | 4 [2 - 5] | 0.82 |
|  |  |  |  |
| *Hospital setting (%)* |  |  | 0.72 |
| Large metropolitan areas | 93.6 | 93.3 |  |
| Small metropolitan areas | 5.9 | 5.8 |  |
| Not metropolitan | 0.5 | 0.8 |  |
|  |  |  |  |
| *Hospital teaching status (%)* |  |  | 0.86 |
| Teaching | 93.6 | 93.3 |  |
| Non-teaching | 6.4 | 6.7 |  |
|  |  |  |  |
| *Bed size (%)* |  |  | 0.01 |
| Small | 7.0 | 13.9 |  |
| Medium | 14.7 | 16.5 |  |
| Large | 78.3 | 69.6 |  |
|  |  |  |  |
| *High-volume status (%)* | 48.5 | 47.5 | 0.81 |
|  |  |  |  |
| Malignant disease (%) | 73.3 | 74.6 | 0.48 |
|  |  |  |  |
| *Comorbidities (%)* |  |  |  |
| Cancer, metastatic | 16.0 | 18.9 | 0.06 |
| Cardiac arrhythmia | 32.5 | 34.5 | 0.16 |
| Chronic liver disease | 6.1 | 5.1 | 0.12 |
| Chronic lung disease | 21.4 | 19.7 | 0.14 |
| Coagulopathy | 7.4 | 7.7 | 0.68 |
| Congestive heart failure | 4.1 | 4 | 0.86 |
| Diabetes | 22.0 | 19.2 | 0.02 |
| End stage renal disease | 5.4 | 6.7 | 0.16 |
| Hypertension | 58.3 | 58.0 | 0.63 |
| Neurologic disorder | 4.5 | 5.1 | 0.28 |
| Obesity | 21.9 | 17.0 | <0.001 |
| Peripheral vascular disease | 5.9 | 5.0 | 0.47 |
| Pulmonary hypertension | 2.4 | 2.4 | 1.00 |
