# Supplementary material for: Association of hospital volume and operative approach with clinical and financial outcomes of elective esophagectomy in the United States
Source: PLoS One. 2024 Jun 14;19(6):e0303586. doi: 10.1371/journal.pone.0303586 (PMC11178205; doi:10.1371/journal.pone.0303586)
Supplement: S4 Table — (DOCX) [file pone.0303586.s004.docx]

**Supplemental Table 4:** Unadjusted perioperative outcomes for laparoscopic or thoracoscopic versus robotic esophagectomy; TIA, transient ischemic attack; pLOS, postoperative length of stay; IQR, interquartile range

|  | **Laparoscopic/**  **Thoracoscopic**  **(n = 6,954)** | **Robotic**  **(n = 3,922)** | **p-value** |
| --- | --- | --- | --- |
| In-Hospital Mortality (%) | 2.1 | 2.2 | 0.86 |
|  |  |  |  |
| **Major Complications (%)** |  |  |  |
| Stroke/TIA | 0.2 | 0.3 | 0.30 |
| Prolonged ventilation | 3.6 | 3.3 | 0.60 |
| Acute renal failure requiring dialysis | 7.4 | 6.8 | 0.50 |
| Reoperation | <0.1 | <0.1 | 0.70 |
|  |  |  |  |
| **Resource Utilization** |  |  |  |
| pLOS (days, median, IQR) | 8 [6 - 11] | 8 [6 - 11] | 0.15 |
| Costs ($1,000s, median, IQR) | 43.0 [27.9 - 62.3] | 41.9 [29.4 - 64.3] | 0.81 |
| 30-day readmission (%) | 12.3 | 14.0 | 0.10 |
