# Supplementary material for: Association of hospital volume and operative approach with clinical and financial outcomes of elective esophagectomy in the United States
Source: PLoS One. 2024 Jun 14;19(6):e0303586. doi: 10.1371/journal.pone.0303586 (PMC11178205; doi:10.1371/journal.pone.0303586)
Supplement: S5 Table — (DOCX) [file pone.0303586.s005.docx]

**Supplemental Table 5:** Adjusted results of robotic approach on clinical outcomes and resource use following esophagectomy as compared to laparoscopic or thoracoscopic approaches; AOR, adjusted odds ratio; CI, confidence interval; β, beta coefficient; TIA, transient ischemic attack; pLOS, postoperative length of stay

|  | **AOR or β Coefficient** | **95% CI** | **p-value** |
| --- | --- | --- | --- |
| In-Hospital Mortality (AOR) | 1.10 | 0.72, 1.67 | 0.65 |
|  |  |  |  |
| **Major Complications (AOR)** |  |  |  |
| Stroke/TIA | 1.63 | 0.56, 4.72 | 0.37 |
| Prolonged ventilation | 0.99 | 0.71, 1.38 | 0.96 |
| Acute renal failure requiring dialysis | 0.98 | 0.75, 1.25 | 0.85 |
| Reoperation | 0.59 | 0.06, 5.34 | 0.64 |
|  |  |  |  |
| **Resource Utilization** |  |  |  |
| pLOS (β, days) | -0.38 | -1.17, +0.42 | 0.35 |
| Costs (β, $1,000s) | 3.97 | 0.8, 7.1 | 0.01 |
| 30-day readmission (AOR) | 1.17 | 0.98, 1.40 | 0.08 |
